# Acceleration and Deceleration Capacity of Fetal Heart Rate in an *In-Vivo* Sheep Model

**DOI:** 10.1371/journal.pone.0104193

**Published:** 2014-08-20

**Authors:** Massimo W. Rivolta, Tamara Stampalija, Daniela Casati, Bryan S. Richardson, Michael G. Ross, Martin G. Frasch, Axel Bauer, Enrico Ferrazzi, Roberto Sassi

**Affiliations:** 1 Dept. of Computer Science, Università degli Studi di Milano, Milan, Italy; 2 Unit of Prenatal Diagnosis, Institute for Maternal and Child Health, IRCCS Burlo Garofolo, Trieste, Italy; 3 Dept. of Woman, Mother and Neonate, Buzzi Children’s Hospital, Biomedical and Clinical Sciences School of Medicine University of Milan, Milan, Italy; 4 Dept. of Obstetrics and Gynecology, Western University, London, Ontario, Canada; 5 Dept. of Obstetrics and Gynecology, LA BioMed at Harbor-UCLA Med. Ctr., Torrance, California, United States of America; 6 CHU Ste-Justine Research Center, Departments of Obstetrics-Gynaecology and Neurosciences, Université de Montréal, QC, Canada; 7 Animal Reproduction Research Centre (CRRA), University of Montreal, St-Hyacinthe, QC, Canada; 8 Dept. of Cardiology, Munich University Clinic, Ludwig-Maximilians University, Munich Germany and DZHK (German Centre for Cardiovascular Research); University of Iowa Carver College of Medicine, United States of America

## Abstract

**Background:**

Fetal heart rate (FHR) variability is an indirect index of fetal autonomic nervous system (ANS) integrity. FHR variability analysis in labor fails to detect early hypoxia and acidemia. Phase-rectified signal averaging (PRSA) is a new method of complex biological signals analysis that is more resistant to non-stationarities, signal loss and artifacts. It quantifies the average cardiac acceleration and deceleration (AC/DC) capacity.

**Objective:**

The aims of the study were: (1) to investigate AC/DC in ovine fetuses exposed to acute hypoxic-acidemic insult; (2) to explore the relation between AC/DC and acid-base balance; and (3) to evaluate the influence of FHR decelerations and specific PRSA parameters on AC/DC computation.

**Methods:**

Repetitive umbilical cord occlusions (UCOs) were applied in 9 pregnant near-term sheep to obtain three phases of MILD, MODERATE, and SEVERE hypoxic-acidemic insult. Acid-base balance was sampled and fetal ECGs continuously recorded. AC/DC were calculated: (1) for a spectrum of *T* values (*T* = 1÷50 beats; the parameter limits the range of oscillations detected by PRSA); (2) on entire series of fetal RR intervals or on “stable” series that excluded FHR decelerations caused by UCOs.

**Results:**

AC and DC progressively increased with UCOs phases (MILD vs. MODERATE and MODERATE vs. SEVERE, p<0.05 for DC 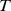 = 2–5, and AC 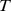 = 1–3). The time evolution of AC/DC correlated to acid-base balance (0.4<
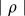
<0.9, p<0.05) with the highest 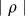 for 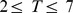. PRSA was not independent from FHR decelerations caused by UCOs.

**Conclusions:**

This is the first *in-vivo* evaluation of PRSA on FHR analysis. In the presence of acute hypoxic-acidemia we found increasing values of AC/DC suggesting an activation of ANS. This correlation was strongest on time scale dominated by parasympathetic modulations. We identified the best performing 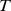 parameters (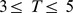), and found that AC/DC computation is not independent from FHR decelerations. These findings establish the basis for future clinical studies.

## Introduction

Labor exposes the fetus to repetitive transient hypoxic stress resulting from uterine contractions and/or umbilical cord compression. Monitoring of the fetal wellbeing and detection of fetal distress during labor are of crucial importance to timely identify hypoxia and to avoid pathologic acidemia. Fetal heart rate (FHR) analysis by cardiotocogram (CTG) is widely used for fetal surveillance in labor. It is characterized by high sensitivity but low specificity for fetal acidemia. After forty years of use, its role in decreasing perinatal mortality or cerebral palsy, despite a marked increase in the rate of operative deliveries, is still controversial [1]. The additional analysis of ST-waveforms on fetal electrocardiograms (ECG) reduced the number of instrumental vaginal deliveries for fetal distress [2]. Nevertheless, it did not solve definitively the issue of fetal acidemia detection [3]. Direct measurement of pH or lactate concentration in humans during partum is feasible by fetal scalp sampling, although this procedure is not universally accepted as a standard of care, and, occasionally, may lead to complications [4]. Thus, there is a need for further advances in fetal monitoring to timely identify fetal hypoxia and acidemia.

Power spectral analysis has been proposed as a method to quantify FHR variability. Several studies have demonstrated changes in power spectrum in relation to fetal hypoxia and/or acidemia during labor [5]–[7]. As with many other composite signals, FHR is generated by a non-stationary system influenced by internal and external perturbations that alter its behavior. Moreover, the phase de-synchronizations due to abrupt changes in the system (as ventricular ectopic beats, maternal uterine contractions, and others), miss-detected beats and signal losses determines a quasi-periodic behavior that limits the application of spectral analysis [8].

Bauer *et al.* [9] introduced a method called phase-rectified signal averaging (PRSA) that emphasizes quasi-periodic oscillations masked by unrelated non-stationary elements in the signal, noise or artifacts. The PRSA series can be employed to quantify the “average acceleration capacity” (AC) and “average deceleration capacity” (DC) of the signal. When applied to heart rate, AC and DC may represent an indirect integrated quantification of the activities of the sympathetic and parasympathetic autonomic systems [10]. The utility of DC was proved in adult cardiology: it is a better predictor of survival in adults who experienced a myocardial infarction [11]. Studies on intrauterine fetal growth restriction showed that PRSA is superior to short term variability in discriminating intrauterine growth restricted fetuses from controls. Nevertheless, those studies used signals obtained indirectly (by CTG [12], [13] or by trans-abdominal ECG recorder [14], respectively), and assessed non-laboring women.

This is the first validation of the PRSA method in a fetal *in vivo* near-term pregnant sheep model. The aim of the study was to investigate changes in AC and DC in response to fetal hypoxia and acidemia, and to evaluate their correlation with acid-base biomarkers. For PRSA analysis, either the whole fRR signal was considered or the segments free of FHR decelerations imposed by umbilical cord occlusions (UCOs). Moreover, specific PRSA parameters were varied (in particular the time scale over which AC and DC are computed).

## Method

### 1. Animal model

Nine near term pregnant sheep were deployed as *in vivo* model. Animal care followed the guidelines of the Canadian Council on Animal Care and was approved by the University of Western Ontario Council on Animal Care. The dataset was previously described [15], [16]. Briefly, after a period of rest (BASELINE), a 1-minute periodical mechanical compression of the ovine fetus’ umbilical cord was continuously alternated with a 1.5 minutes recovery. Three levels of occlusion strength, from partial to complete, were designed: mild (MILD, 60 minutes), moderate (MODERATE, 60 minutes) and complete (SEVERE, ∼2 hours or until pH<7.00 was reached). Physiologic results from these and additional animals have been previously reported [15]–[18].

Electrodes implanted into the left supra-scapular muscles, in the muscles of the right shoulder, and in the cartilage of the sternum of the fetus were used to measure the ECG which was digitized at 1000 Hz. Fetal blood samples were collected with intervals of 20 minutes to quantify the values of pH, lactate and base deficit (hereafter referred to as “biomarkers”). The severe phase of UCOs was stopped when the pH dropped below 7.0. Then, a recovery phase (RECOVERY) concluded the protocol. ECGs were automatically analyzed to obtain the sequence of fetal RR intervals. Due to the long time span over which the data were collected, heart beat misdetections were common in ECG, especially during the actual umbilical compression. We considered suitable for further analysis only those sheep (7 out of 9) which had more than 90% of correctly located beats during MODERATE and SEVERE phases (gaps in the series were less than 10% of the total time). During the MILD phase, miss-detected beats were less than 10% in the entire population.

### 2. Phase-rectified signal averaging

PRSA provides an estimate of the autonomic regulation of the FHR even when phase de-synchronizations due to abrupt changes in the system, miss-detected beats and signal losses are present [9]. Briefly, a set of anchor points is determined on the fRR series: each time point 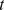 that satisfies the following criterion is inserted into the anchor points’ list (deceleration):
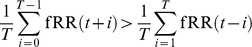



A window of length 2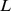 is centered on each anchor point (the anchor point is at position 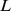
+1). Then, the windows are aligned and averaged, obtaining the PRSA series. Finally, the PRSA series is used to compute the DC with:
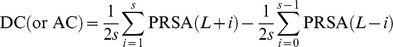



It is worth noting that this expression is substantially equivalent to a wavelet transform (Haar wavelet) of the PRSA series, evaluated at scale 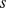 and location 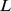
+1.

Three parameters, 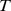, 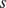 and 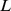, need to be specified. 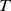 sets the number of points of the low-pass moving average filter employed before the detection of anchor points. It is an upper frequency limit for the periodicities that can lead to the selection of anchor points by PRSA (*i.e.*, the 3 dB pass-band of the filter ends approximately at 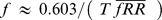 Hz, where 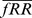 is the average fetal RR interval in seconds). 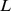 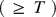 determines the extension of the PRSA series. In principle, 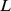 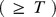 needs to be larger than the period of the slowest oscillation to be detected with PRSA; however, when computing AC or DC, it suffices to be as large as 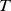. Finally, the scale s selects the oscillations in the PRSA series that most affect AC and DC. Approximately, using a Haar wavelet, the scale s corresponds to the frequency 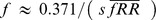 Hz. In this study, the scale s is taken to be equal to 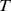. This is not mandatory (*i.e.,* in [11] 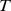 = 1 and 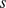 = 2 were found to be optimal for prediction of mortality after myocardial infarction in adults), but in line with two previous studies employing PRSA on fRR series [12], [13]. Using 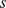 = 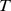 we avoided the need of optimizing a further parameter. We leave this effort to further studies.

An identical procedure is used to compute AC, but after employing a different criterion for selecting the anchor points:
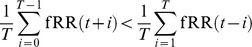



In this framework, DC values are positive and AC values are negative. However, for simplicity, in this work we chose to consider only their absolute values, so that both are positive.

### 3. Preprocessing

Fetal RR intervals greater than 1500 ms (40 bpm) were labeled as artifacts and substituted with an equivalent number of beats (calculated dividing the length of each artifact by the median of the 20 nearby fRR samples). These reconstructed samples were not used as anchor points in the PRSA analysis; however they contributed to the selection of nearby anchor points. Furthermore, each fRR interval that exceeded the preceding one by more than 20% was excluded from the anchor points’ lists.

### 4. Protocol of the study

We analyzed fRR series obtained during the last 30 minutes of each UCO phase. The rationale for this choice was that, although hypoxic-acidemia induced by UCOs grew gradually over time throughout the UCOs, it was respectively maximal within the last part of each UCO phase. Consequently, time-matched biomarkers’ values were those collected with the last blood sample in each UCO phase, so that the number of samples was equal for each individual across the cohort avoiding a possible statistical bias in correlation coefficients (data available in Table S1).

AC and DC were determined independently for each of the UCO phases by PRSA analysis. We checked that more than 150 anchor points were available in each UCO phase. The computation was performed for values of 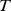 in the range 1–50 with 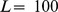. The linear correlation between AC/DC and biomarkers’ values was assessed by Spearman’s correlation coefficient (p<0.05 was considered significant).

**Figure 1:**
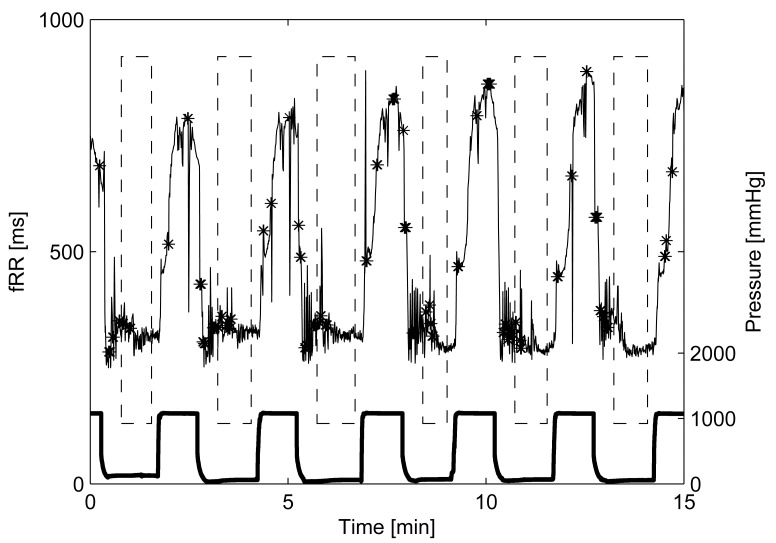
Example of fRR series and umbilical cord occlusions (UCOs) pressure signal (bottom bold line). Dashed boxes emphasize stable fRR intervals (without artifacts and UCO-induced decelerations). Black stars mark artifacts or reconstructed fRR samples, excluded from being anchor points in the PRSA analysis.

**Figure 2:**
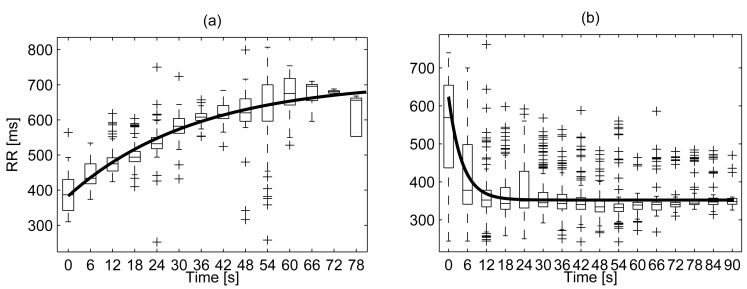
Example of estimation of the fRR time constants during cord occlusion and subsequent recovery. Panel (a): fRR during mechanical cord occlusion. The data belongs to the fetus of sheep #3 (Table 1). Panel (b): fRR during recovery after UCO for the same case. Box plots summarize the values of fRR at a given time distance from the occlusion (or its release). The model prediction is reported with a bold line. The vertical lines delimit the “whiskers” and the + signs the outliers.

UCO alters significantly the FHR, and the influence of FHR on AC/DC is still matter of investigation. For this reason, a second PRSA analysis was performed after excluding FHR decelerations due to UCO, *i.e.*, macro oscillations (Figure 1). “Stable” FHR intervals employed were located using the pressure signal applied on the umbilical cord during occlusion (Figure 1). At the beginning of each occlusion, fRR decreased progressively to quickly recover when pressure was released. The time constants of the fRR during SEVERE cord occlusion (“stim”) and recovery (“rec”) were estimated, for each sheep, by fitting the exponential model:
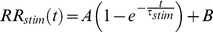


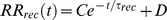
where 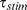 and 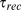 are two time constants (*A*, *B*, *C* and *D* are scalars necessary for the fitting but not further considered in this study). Levenberg-Marquardt least-square estimation was used to estimate the parameters of the model for each sheep (median 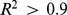). An example is reported in Figure 2 while Table 1 lists the values of 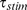 and 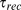. The two sets of time constants were largely different (p<0.05, paired Wilcoxon signed rank test) suggesting that the recovery of the HR is far quicker than the onset of the deceleration.

**Table 1:** Time constants of the fRR-response to UCO (in seconds).

SHEEP	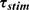	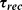
**#1**	9.63	6.46
**#2**	46.17	5.22
**#3**	34.56	4.30
**#4**	14.22	3.38
**#5**	34.65	7.88
**#6**	12.46	4.36
**#7**	11.76	5.61
**Mean**	23.35	5,32
**Median**	14.22	5.22
**IR**	12.11–34.61	4.33–6.03

We considered stable (free from FHR decelerations) those intervals spanning 30 s after the end of each occlusion and ending at the beginning of the next one. The value of 30 s was selected to be larger than 3 times the longest recovery time, *i.e.*, 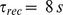, so that fRR is substantially back to the baseline value. By definition, stable baseline intervals between decelerations were short (at most 1 minute each once removed artifacts): to explore larger value of 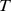 they were concatenated (data available in Table S1).

**Figure 3:**
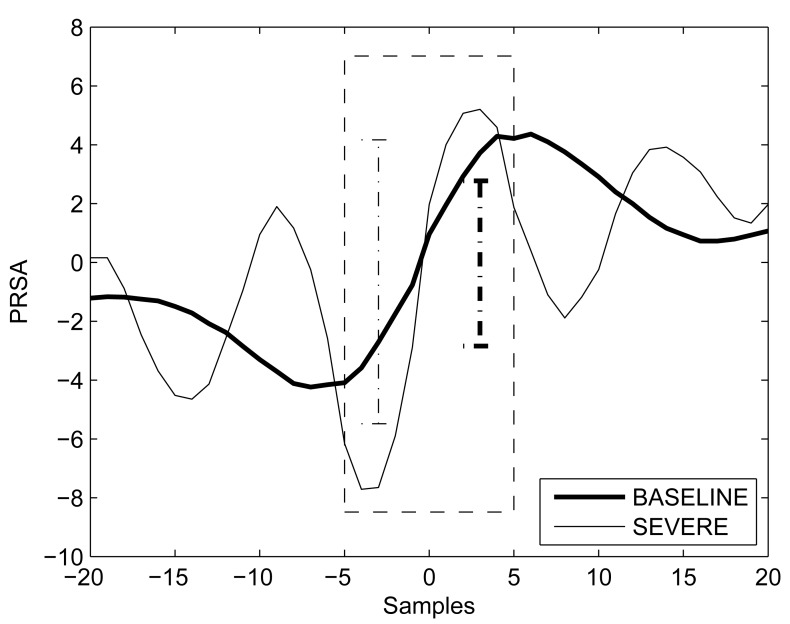
PRSA curve (DC) for a single case during BASELINE (bold line) and SEVERE UCO (PRSA’s mean value was removed). The analysis was performed on stable (concatenated baseline free of FHR decelerations segments) fRR intervals. The dashed box emphasizes samples used for computing DC (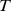 = 5). Vertical dotted bars depict DC in the two cases considered.

Summarizing, for each UCO phase two sets of AC/DC values were obtained: 1) from the entire fRR series (entire fRR); and 2) from stable baseline and free of FHR decelerations fRR intervals extracted from the fRR series and then concatenated (stable fRR). Two examples of PRSA curves are shown in Figure 3.

A paired Wilcoxon signed rank test was employed to compare AC/DC values between: BASELINE and MILD; MILD and MODERATE; MODERATE and SEVERE. A Bonferroni correction for multiple comparisons was applied. A non-parametric test was preferred due to the small number of samples.

## Results

Table 2 contains the values of the biomarkers (pH, lactate and base deficit) for each protocol phase. As expected from the protocol’s design, there was an increasing trend from BASELINE to SEVERE UCOs.

**Table 2:** Median values of pH, lactate and base deficit according to protocol phases.

	BASELINE	MILD	MODERATE	SEVERE
**pH**	7.34 (7.34, 7.37)	7.33 (7.31, 7.34)	7.28 (7.24, 7.30)	6.98 (6.96, 7.07)
**Lactate**	1.60 (1.33, 1.85)	1.65 (1.40, 2.05)	3.80 (2.68, 4.60)	11.40 (5.20, 12.43)
**Base deficit**	1.08 (0.31, 3.28)	0.29 (−2.10, 1.06)	−2.46 (−3.85, −1.17)	−14.07 (−15.34, −12.64)

Interquartile ranges are reported within brackets. Lactate and base deficit are shown in mEq/L.

### 1. AC and DC change during protocol phases

Values of AC and DC followed a similar growing trend from BASELINE to SEVERE UCOs. Table 3 reports median AC and DC values for each protocol phase and for several 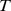 values (PRSA was computed on the entire fRR signal). Differences between phases varied according to the value of 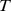 employed. The list of 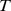 values for which a statistically significant difference was found between successive phases (p<0.05) is contained in Table 4. Summarizing, as the hypoxic-acidemia progressed, DC (for 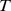 = 2 to 5) and AC (for 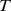 = 1 to 3) were different between MILD and MODERATE and then between MODERATE and SEVERE. However, MILD phase was not distinguishable from BASELINE. Parameters computed with higher values of 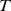 were different between MILD and MODERATE only.

**Table 3:** AC and DC absolute median values (in ms) when considering the entire signal (including UCO-induced FHR decelerations).

AC	BASELINE	MILD	MODERATE	SEVERE
**T = 2**	2.30 (1.55,2.51)	1.92 (1.56,2.26)	4.32 (3.40,6.14)	6.57 (4.21,8.18)
**T = 4**	2.77 (2.35,3.56)	2.56 (2.25,3.91)	5.33 (4.85,7.12)	7.01 (5.19,8.71)
**T = 6**	3.27 (2.99,3.93)	3.14 (2.78,4.45)	6.13 (5.89,7.77)	6.84 (6.30,8.45)
**T = 10**	4.07 (3.60,4.24)	4.06 (3.59,4.82)	8.09 (7.07,8.82)	6.40 (5.82,9.02)
**T = 20**	4.58 (4.12,4.66)	5.04 (4.75,5.50)	10.92 (8.96,11.21)	7.81 (7.02,10.60)
**DC**	**BASELINE**	**MILD**	**MODERATE**	**SEVERE**
**T = 2**	2.78 (1.93,3.79)	2.26 (2.05,3.19)	4.91 (3.72,5.85)	7.21 (4.36,7.55)
**T = 4**	3.00 (2.47,4.06)	2.86 (2.65,4.20)	6.19 (4.93,7.48)	8.92 (5.89,9.61)
**T = 6**	3.29 (2.98,4.42)	3.49 (3.26,4.60)	6.62 (6.07,8.45)	9.55 (7.56,10.14)
**T = 10**	3.74 (3.46,4.79)	4.44 (4.19,5.18)	7.69 (7.37,9.86)	10.40 (9.30,10.82)
**T = 20**	4.21 (3.74,4.96)	5.61 (5.48,5.88)	9.45 (8.87,10.22)	12.24 (9.65,14.66)

Interquartile ranges are reported within brackets. Any AC or DC value (for MILD, MODERATE and SEVERE) is statistically different from the corresponding one in Table 3 (p<0.05).

**Table 4:** Ranges of 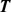 in which a significant difference between two phases was found (Wilcoxon signed rank test, p<0.05).

	BASELINE	MILD	MODERATE	SEVERE
**BASELINE**	-	none	-	-
**MILD**	-	-	any	-
**MODERATE**	-	-	-	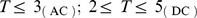

PRSA was performed on the entire signal (including UCO-induced FHR decelerations).

When repeated on stable fRR intervals, the results were substantially confirmed even though less markedly, as reported in Table 5 and Table 6. Specifically, while SEVERE was distinguishable from MODERATE for 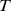 in the range 3 to 5, MILD and MODERATE were significantly different only for large values of 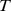. However, for 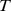
>25, anchor points were selected using fRR samples coming from two consecutive recovery periods, and, thus, the results are less reliable.

**Table 5:** AC and DC absolute median values (in ms) when considering stable (concatenated baseline segments free of FHR decelerations) fRR intervals.

AC	BASELINE	MILD	MODERATE	SEVERE
**T = 2**	2.31 (1.55,2.53)	1.61 (1.17,2.11)	2.18 (1.75,2.85)	3.99 (2.10,4.34)
**T = 4**	2.77 (2.36,3.52)	2.14 (1.68,2.67)	2.79 (2.47,3.58)	4.45 (3.10,5.29)
**T = 6**	3.26 (3.00,3.87)	2.49 (2.12,2.96)	3.41 (2.95,4.20)	4.81 (3.49,5.22)
**T = 10**	4.07 (3.61,4.24)	3.01 (2.46,3.48)	4.31 (3.31,5.46)	3.99 (3.84,5.26)
**T = 20**	4.53 (4.13,4.68)	3.34 (2.73,4.41)	5.43 (3.92,6.25)	5.36 (4.42,5.94)
**DC**	**BASELINE**	**MILD**	**MODERATE**	**SEVERE**
**T = 2**	2.79 (1.95,3.78)	2.00 (1.54,2.95)	2.68 (2.28,3.13)	4.18 (2.82,4.44)
**T = 4**	2.99 (2.50,4.03)	2.37 (1.85,2.96)	3.11 (2.89,4.10)	4.87 (3.57,5.25)
**T = 6**	3.27 (3.01,4.36)	2.79 (2.37,3.26)	3.61 (3.28,5.13)	4.81 (3.63,6.11)
**T = 10**	3.73 (3.49,4.70)	3.22 (2.81,4.11)	4.60 (3.67,6.48)	4.83 (4.49,6.29)
**T = 20**	4.20 (3.77,4.86)	3.82 (3.55,5.10)	5.55 (4.53,7.59)	6.35 (4.99,7.45)

Interquartile ranges are reported within brackets. Any AC or DC value (for MILD, MODERATE and SEVERE) is statistically different from the corresponding one in Table 2 (p<0.05).

**Table 6:** Ranges of 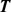 in which a significant difference between two phases was found (Wilcoxon signed rank test, p<0.05).

	BASELINE	MILD	MODERATE	SEVERE
**BASELINE**	-	none	-	-
**MILD**	-	-	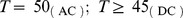	-
**MODERATE**	-	-	-	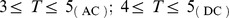

PRSA was performed on stable (concatenated baseline segments and free of FHR decelerations) fRR intervals.

Furthermore, AC/DC values, computed on a window of 5 minutes immediately after the end of the SEVERE phase, were not statistically different from those during the BASELINE phase (p>0.05).

Finally, during UCO the values of AC/DC computed on the entire signals (Table 3) were significantly different from those obtained on stable series (Table 5), and this was true for each value of 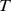 (p<0.05).

### 2. Correlations with acid base balance

**Figure 4:**
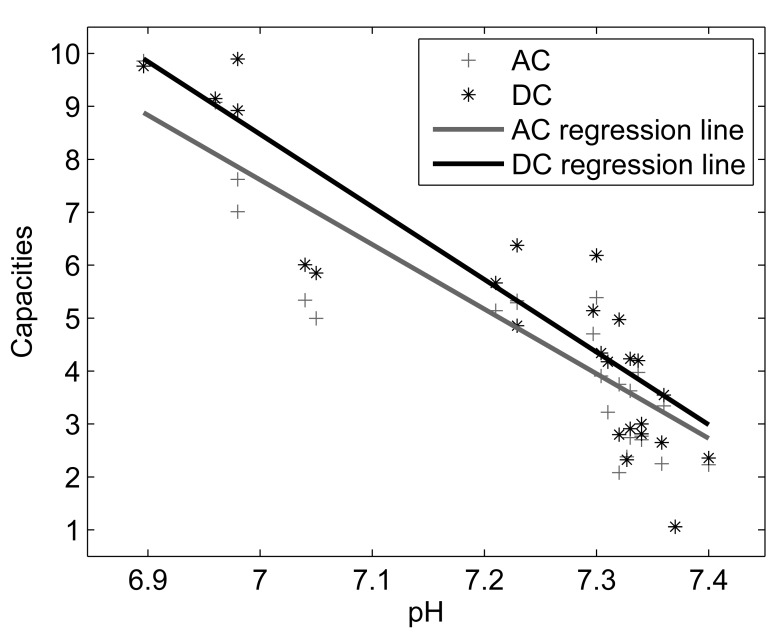
Relation between AC and DC with pH for 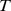 = 4 (entire fRR series) .

**Figure 5:**
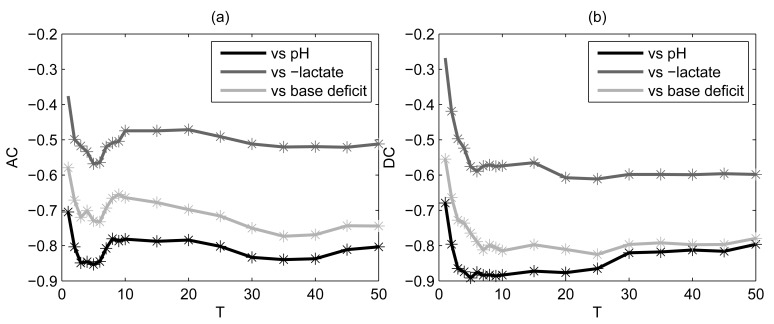
Spearman’s correlation coefficients between AC (a) and DC (b) and each acid-base balance biomarker. PRSA was performed on the entire fRR series. Stars refer to significant p values (p<0.05). Lactate values were multiplied by −1.

A significant Spearman’s correlation coefficient between AC/DC (computed on the entire fRR series) and each biomarker of acid base balance was found for a large range of 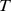 values (Figure 4 and Figure 5). However, maximal correlation (0.40<
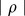
<0.90; p<0.05) was for 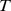 in the interval 2 to 6, and was stronger for DC. Both AC and DC correlated stronger to pH and base deficit, than to lactate concentration. For instance, considering 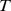 = 4, we found a negative correlation among AC (its absolute value) and pH (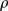 = −0.85; p<0.05), base deficit (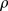 = −0.70; p<0.05), and a positive correlation with lactate (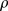 = 0.53; p<0.05). Similarly, we found a negative correlation among DC and pH (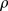 = −0.87; p<0.05), base deficit (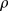 = −0.74; p<0.05), and a positive correlation with lactate (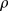 = 0.52; p<0.05).

**Figure 6:**
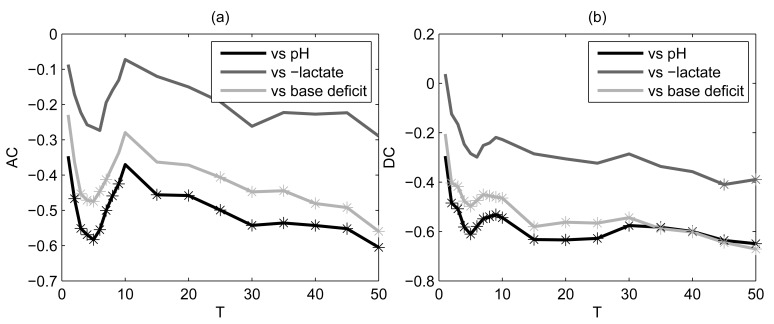
Spearman’s correlation coefficients between AC (a) and DC (b) and each acid-base balance biomarker. PRSA was performed on stable (concatenated baseline and free of FHR decelerations) baseline fRR intervals. Stars refer to significant p values (p<0.05). Lactate values were multiplied by **−**1.

When excluding decelerations imposed by UCOs, a significant correlation was found for a narrower range of 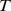 values (Figure 6), the range was wider for DC than AC). However, these values matched those for which the correlation coefficients were maximal in the previous analysis.

## Discussion

The principal findings of the study are: 1) AC and DC increase with worsening acidemia; 2) AC and DC correlate to acid base balance observed at different phases of hypoxic-acidemia; 3) PRSA computed with 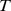 = 2 to 5 best enhances differences among protocol phases (this is particularly true for SEVERE cord occlusions), leading to the highest correlation between AC or DC with biomarkers; and 4) considering only stable fRR segments (concatenated baseline segments of FHR between decelerations) or the entire fRR series led to different results (AC/DC values obtained on stable fRR segments were smaller on average). Thus, FHR decelerations, even if low frequency, induce changes in the PRSA series and, consequently, in AC and DC.

### 1. AC and DC increase with worsening acidemia

AC and DC identify different behavior of the FHR during acceleration and deceleration, respectively. Nevertheless, it would be too simplistic to consider AC only as an expression of sympathetic modulation and DC as an expression of parasympathetic modulation. Rather, both AC and DC result from the interaction of parasympathetic and sympathetic component, and represent an integration of several input signals such as chemoreceptor, baroreceptor and others. The two components of the autonomic nervous system (ANS) operate at different frequency scales: sympathetic component in low frequency domain, while parasympathetic both in low and high frequency domain, respectively. In PRSA computation, the 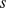 = *T* parameter determines an upper frequency limit for the periodicities that mostly influence AC and DC [9]. For 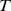 = 1 high frequencies dominate the computation. At contrary, increasing values of 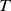 will progressively also emphasize the contribution of low frequency components.

When looking at the absolute values of AC and DC we observed a clear increasing trend with worsening acidemia. Indeed, during SEVERE UCOs (*i.e.,* severe acidemia), the AC and DC were maximal with respect to BASELINE. This trend was present when analyzing the whole fRR series and for a large range of 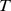 values suggesting the activation of both sympathetic and parasympathetic component during progressive acidemia.

However, only a smaller range of 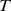 (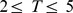) was able to differentiate MODERATE vs SEVERE phase. The same was true for 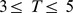 when excluding FHR decelerations. This would suggest that, for higher degree of acidemia, the frequencies dominated by parasympathetic component become predominant. These findings are in agreement with other reports that evaluated ANS response to hypoxia, but with different methodologies: 1) Frasch *et al*, in the same population of 7 near-term pregnant sheep employed for this work, reported that the root mean square of successive differences (RMSSD, a time-domain index mainly influenced by the vagal activity [19]) has the most pronounced changes during acidemia [20]; 2) Siira *et al.* found that in an acute phase of hypoxia, without acidemia, there is an activation of sympathetic system, while, when acidemia occurs, the vagal influence increases [5].

The activation of ANS by initial and acute hypoxia most likely constitutes a first line adaptive response, and results in a more pronounced FHR modulation and a larger cardio-vascular response. Indeed, in experiments on fetal lambs, acute fetal hypoxia led to increased FHR variability representing a sign of adequate fetal compensatory response [21]. Moreover, the predominant involvement of vagal tone has been associated to a more efficient modulation of ANS [22]. In fact, in the presence of acute hypoxia, the reduction in FHR (*i.e.*, deceleration) is thought to be protective for the fetus, because it reduces myocardial work and oxygen consumption [23], and is mediated by the chemoreceptors via the parasympathetic branch. Nevertheless, if there is a prolonged hypoxic insult and overwhelming acidemia, parasympathetic activity decreases [5], causing reduced FHR variability [24]. In fact, when the vagal regulation becomes inadequate, some of the adaptive mechanisms (such as chemoreceptor-mediated circulatory adaptation) might fail causing fetal brain damage, and ultimately fetal death.

Interestingly, our data did not show the final fall in parasympathetic activity before the pH reached the predefined threshold, most likely due to the acute nature of the insult. This was confirmed by the rapid recovery (Table 1 and Figure 2) that each animal showed both for acid-base balance and PRSA parameters [15].

### 2. AC and DC correlate with acid-base balance

We found that AC and DC correlate to the biomarkers of acidemia, and this correlation is significant both for fRR series that include and exclude FHR decelerations. Interestingly, the correlation was stronger for pH and base deficit, and to a lesser extent for lactate concentration. This finding can be explained by the fact that both pH and base deficit are strong stimulators of chemoreceptors which are highly sensitive to the presence of hypoxemia, and, consequently, influence FHR modulation [25].

Moreover, we found that the correlation with acidemia was maximal for low values of 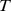. This would suggest that, in the presence of acute hypoxic insult, the low frequencies dominated by parasympathetic branch are more responsive to the changes of acid base balance. There are studies that evaluated the correlation between FHR variability assessed by spectral analysis and acid base balance, either during labor [5], or at birth [6], [26]. Although, all studies confirmed that hypoxia and acidemia have a direct effect on FHR variability, and thus on ANS, it is difficult to derive a common line for all studies because of profound methodological differences [7]. Nevertheless, our findings are in agreement with those by Siira *et al.*, who found a correlation between high frequency bands at power spectral analysis of FHR variability and pH obtained by fetal scalp blood sampling during labor [5].

### 3. Changes in AC and DC varying the parameter 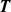


The significance of the parameter 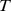 has been explored in adult cardiology, and a 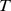 of 1–2 has been found as the best value [10] (when 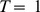, 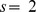 might be preferred [11]). Although some studies applied PRSA to FHR [12]–[14], the impact of changing 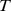 when PRSA is applied to FHR has not been reported. We found that 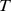 value in interval 2–5 best enhances the differences between progressive cord occlusion phases (worsening acidemia). Similarly, when evaluating the correlation with acid-base biomarkers, the best correlation was observed for 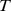 value in the range 2–6. Very recent analyses in adult cardiology also showed that application of a larger 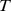 makes PRSA more robust to artifacts and noise [27]. Thus, we suggest this time scale for an effective computation of PRSA analysis, when detecting hypoxic-acidemic events in laboring fetuses. However, the small sample size employed requires further studies.

### 4. Excluding FHR decelerations in PRSA computation

Next, we wanted to evaluate if there are significant differences between stable intervals of fRR series, and the entire fRR series. The rationale was the fact that abrupt perturbations, such as uterine contractions, or in this case UCOs, may lead to a phase de-synchronization in the fRR series. Interestingly, we found significant differences between AC and DC computed on the entire signal or on stable fRR signal, respectively. This was true even for small values of 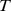, and, thus, for high order frequencies. When interpreting these findings it has to be taken into account that: 1) the frequency content of macro oscillations is very limited (compared with those of other components of the fRR), and, thus, should be filtered out from the series when calculating PRSA; and 2) capacity estimates should be independent from FHR decelerations when ANS regulation does not change (at least for small values of 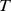
<20). However, our results showed the contrary. There could be two possible explanations. First, during UCOs, not only the mean trend of the series changes but also the beat-to-beat relationships regulated by ANS. Second, PRSA’s amplitude depends on the power of the oscillatory components in the signal, and, thus, AC and DC may be influenced by severe FHR decelerations that determine changes in total spectral power.

The analysis on the stable fRR segments seems to strengthen the first explanation. Moreover, in order to address the issue of total power of the signal we computed the standard deviation of normal-to-normal intervals (SDNN, [21]), a FHR variability measure capturing all cyclic components responsible for the variability in the period of recording and strictly related to the total power of the sequence. SDNN was statistically indistinguishable between any two consecutive phases of the stable segments of fRR intervals (paired Wilcoxon signed rank test, p>0.05; data not shown). Therefore, the total power cannot explain the difference in AC and DC between phases.

To summarize, we found differences in AC/DC between entire and stable fRR series, respectively, that in our data series cannot be explained by the change in total power suggesting a higher order influence of ANS on fRR series during decelerations. Which one of the two phases (*i.e.*, entire or stable fRR series) could be more valuable in a clinical scenario remains to be evaluated.

## Conclusions

In conclusion, our study shows that PRSA-based analysis of FHR variability is a sensitive tool for detecting hypoxia-induced autonomic activations. Overall, we found the evidence of ANS activation in sheep fetus exposed to acute hypoxic-acidemic insult. Such activation was more prominent at mid/high frequencies (which mostly influence PRSA in the range 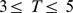), that correspond to a more relevant activation of the parasympathetic branch). Moreover, the PRSA-based measures, AC and DC, were significantly correlated with measures of acidemia with strongest correlation in a range of high frequencies. We evaluated the impact of the parameter 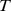 and we offered suggestions for its choice. These findings establish the basis for future clinical studies.

### Limitations and strengths of the study

This is the first validation, in an *in vivo* pregnant sheep model, of PRSA analysis of FHR to detect different acid-base states during worsening hypoxic-acidemia. Studies performed on *in vivo* models (usually pregnant sheep) permit to simulate near-term pregnancies, and repetitive UCOs to mimic uterine contractions. Moreover, the data such as fRR series, pH, base deficit and lactate concentration are obtained directly from the fetus during the entire course of the experiment, increasing the accuracy of the findings.

A limitation of the study is the small sample size. However, it was adequately powered to detect differences of DC and AC at different hypoxic phases.

## Supporting Information

Table S1 The table shows, for each sheep, the absolute median values (in ms) of AC and DC computed for *T* values in the interval 1–50 in each of the phases of the experiment (baseline, mild, moderate and severe phases). The analysis of both the entire signal (including FHR decelerations) and stable fRR intervals (concatenated baseline segments free of FHR decelerations) are reported. Median values of pH, lactate and base deficit collected in each of the protocol phases are also reported for each sheep. Lactate and base deficit are shown in mEq/L.
(XLS)
